# Spinocerebellar ataxia type 8 larger triplet expansion alters histone modification and induces RNA foci

**DOI:** 10.1186/1471-2199-10-9

**Published:** 2009-02-10

**Authors:** I-Cheng Chen, Hsuan-Yuan Lin, Ghin-Chueh Lee, Shih-Huan Kao, Chiung-Mei Chen, Yih-Ru Wu, Hsiu-Mei Hsieh-Li, Ming-Tsan Su, Guey-Jen Lee-Chen

**Affiliations:** 1 Department of Life Science, National Taiwan Normal University, Taipei 116, Taiwan; 2 Department of Neurology, Chang Gung Memorial Hospital, Chang-Gung University College of Medicine, Taipei 105, Taiwan

## Abstract

**Background:**

Spinocerebellar ataxia type 8 (SCA8) involves the expression of an expanded CTG/CAG combined repeats (CR) from opposite strands producing CUG expansion transcripts (ataxin 8 opposite strand, ATXN8OS) and a polyglutamine expansion protein (ataxin 8, ATXN8). The pathogenesis of SCA8 is complex and the spectrum of clinical presentations is broad.

**Results:**

Using stably induced cell models expressing 0, 23, 88 and 157 CR, we study the role of ATXN8OS transcripts in SCA8 pathogenesis. In the absence of doxycycline, the stable ATXN8OS CR cell lines exhibit low levels of ATXN8OS expression and a repeat length-related increase in staurosporine sensitivity and in the number of annexin positive cells. A repeat length-dependent repression of ATXN8OS expression was also notable. Addition of doxycycline leads to 25~50 times more ATXN8OS RNA expression with a repeat length-dependent increase in fold of ATXN8OS RNA induction. ChIP-PCR assay using anti-dimethyl-histone H3-K9 and anti-acetyl-histone H3-K14 antibodies revealed increased H3-K9 dimethylation and reduced H3-K14 acetylation around the ATXN8OS cDNA gene in 157 CR line. The repeat length-dependent increase in induction fold is probably due to the increased RNA stability as demonstrated by monitoring ATXN8OS RNA decay in cells treated with the transcriptional inhibitor, actinomycin D. In cells stably expressing ATXN8OS, RNA FISH experiments further revealed ribonuclear foci formation in cells carrying expanded 88 and 157 CR.

**Conclusion:**

The present study demonstrates that the expanded CUG-repeat tracts are toxic to human cells and may affect ATXN8OS RNA expression and stability through epigenetic and post-transcriptional mechanisms.

## Background

Spinocerebellar ataxia type 8 (SCA8) is a dominantly inherited, slowly progressive neurodegenerative disorder caused by the expansion of CTA/CTG combined repeats (CR) in the ataxin 8 opposite strand (ATXN8OS) gene located on chromosome 13q21 [1]. The reported repeat lengths associated with ataxia vary dramatically, ranging from 68 [2] to >1000 repeats [3]. In the general population more than 99% of the alleles have 16~37 CR [1]. Nevertheless the penetrance of the SCA8 repeat expansion and ataxia is not complete, as expansions do not always segregate with ataxia in families and they are present in rare instances in normal and non-ataxic diseased populations [1,3-7].

The pathogenesis of SCA8 is complex. In addition to a CTG repeat expansion in the ATXN8OS gene, it also involves a CAG repeat expansion in another overlapping gene, ataxin 8 (ATXN8) [8]. In the CTG direction, ATXN8OS expresses non-coding transcripts containing the CUG expansion which overlap with the 5' region of the Kelch-like 1 (KLHL1) transcripts, and in the CAG direction ATXN8 expresses transcripts encoding a nearly pure polyglutamine expansion protein. As a consequence, three plausible mechanisms were proposed for SCA8: RNA gain-of function [9], partial loss of KLHL1 function [10] and polyglutamine expansion protein in the CAG direction [11]. In the present study, we focus on the RNA gain-of function mechanism.

The causative agent for myotonic dystrophy (DM1) is also known to be a CTG expansion in the 3'-UTR of the DMPK gene [12]. The expanded CUG repeat in the DMPK RNA impaired nuclear cytoplasmic transport, resulting in nuclear retention and ribonuclear foci formation [13,14]. In addition, expanded CTG repeats in DM1 alter the adjacent chromatin structure [15] and several proteins bind to CUG repeat-containing RNA [16,17]. Using PC12 neuronal cells expressing the CUG repeat-bearing mRNA, *cis*-effects through the reporter gene and neuronal death after cell differentiation *in vitro *were reported [18]. Expression of a Huntington's disease-like 2 JPH3 transcript with an expanded CUG repeat also resulted in the formation of RNA foci and cell toxicity [19]. Based on these previous studies, we established ATXN8OS stably induced HEK-293 cell lines carrying 0, 23, 88 and 157 CR to investigate the possible epigenetic and post-transcriptional regulations of the ATXN8OS expression.

## Results

### ATXN8OS CR cell lines

**Figure 1:**
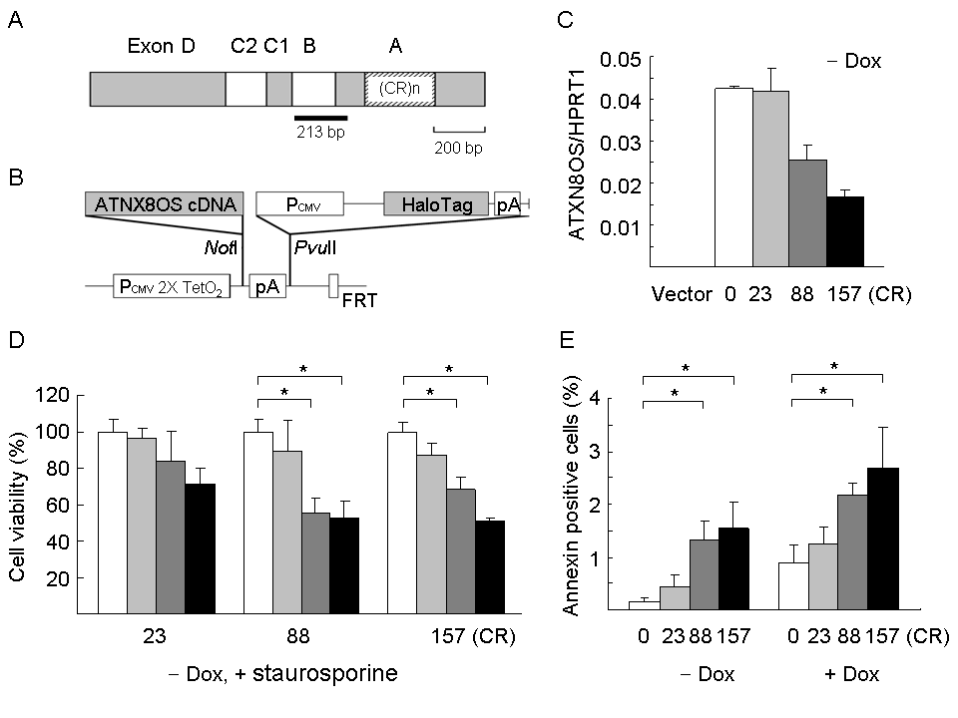
**ATXN8OS 0~157 CR cells**. (A) The ATXN8OS cDNA containing exons D, C2, C1, B and A. The combined repeats (CR)n inside exon A are indicated. The thick black line below cDNA represents a 213-bp fragment spanning exons B to A for ChIP-PCR. (B) The features of pcDNA5/FRT/TO-ATXN8OS plasmids. ATXN8OS cDNA was cloned into the *Not*I site of the pcDNA5/FRT/TO vector and its expression driven by hybrid CMV/TetO_2 _promoter (P_CMV _2X TetO_2_). Also shown is the fragment containing CMV promoter, HaloTag open reading frame and SV40 late poly(A) signal placed at the *Pvu*II site between bovine growth hormone poly(A) signal and Flp recombination target (FRT) site. (C) Real-time PCR quantification of ATXN8OS RNA level relative to endogenous HPRT1 RNA in ATXN8OS CR cells grown without doxycycline. (D) The effect of 0 nM (white bar), 15 nM (gray bar), 30 nM (charcoal gray bar) and 50 nM (black bar) staurosporine on the survival of ATXN8OS 23, 88 and 157 CR cells grown without doxycycline. (E) Annexin V binding in ATXN8OS 0, 23, 88 and 157 CR cells grown with (right) or without (left) doxycycline. Data are represented as the mean ± SD of three independent experiments, each performed in duplicate. The * indicates the difference between the indicated samples are statistically significant (*P *< 0.05).

The pcDNA5/FRT/TO vector and ATXN8OS constructs containing 0, 23, 88 and 157 CR were used to generate ATXN8OS CR cell lines. These cell lines were originated from human embryonic kidney 293 cells, which express many neuron-specific mRNAs [20]. A large body of work on other repeat expansion diseases with similar neuronal pathology using this cell line has been reported [21,22]. The derived ATXN8OS cell lines are isogenic except for the number of CTA/CTG combined repeats. The repeat number in these cell lines was stable (data not shown). ATXN8OS RNA levels were measured by real-time PCR quantification using ATXN8OS-specific probe and primers. The expression of the endogenous ATXN8OS RNA in vector only cell line was too low to be efficiently detected. In the absence of doxycycline, all ATXN8OS CR cell lines expressed low level of ATXN8OS RNA, ranging from 0.017 to 0.042 compared with endogenous HPRT1 (Fig. 1C). A repeat length-dependent repression of ATXN8OS expression is notable. ATXN8OS 88 and 157 CR cells were more sensitive to staurosporine (30 and 50 nM), an external apoptotic stimulus (Fig. 1D). A repeat length related increase in the number of annexin V-positive cells was also observed when the viability of these ATXN8OS CR cell lines was examined. Annexin V binds phosphatidyl serine (PS) located in the plasma membrane. PS is only accessible to annexin V during apoptosis when the PS moves from the inner to the outer plasma membrane, or during necrosis when membrane integrity is lost. In CR cells grown without doxycycline, while the absolute level of cell death was relatively modest, in 88 and 157 CR the number of dying cells was statistically significant amounting to ~3 times that seen in the cells with 23 CR (Fig. 1E, – Dox). In CR cells grown with doxycycline, cell death was also significantly increased, amounting to ~2 times that seen in the cells with 23 CR (Fig. 1E, + Dox).

**Figure 2:**
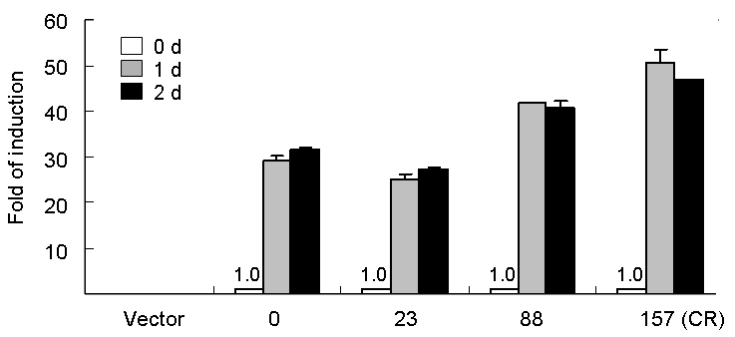
**Expression of ATXN8OS RNA in 0~157 CR and vector only lines**. Real-time PCR quantification was performed after addition of doxycycline for 0~2 days. To calculate the fold of induction, the relative ATXN8OS RNA levels in each CR line at the time of doxycycline addition (0 d) are set as 1.0. Data are represented as the mean ± SD of two independent experiments, each performed in duplicate.

Repeat length-related change in ATXN8OS expression The induction of ATXN8OS RNA levels were further examined in these CR cells (Fig. 2). After induction with doxycycline for 1 and 2 days, the amount of ATXN8OS RNA in 0, 23, 88 and 157 CR cells increased significantly. When the amount of ATXN8OS RNA present at the time of doxycycline addition was set to 100% for each CR cell line, the fold of induction increased with repeat length, with (CR)_n_/fold of induction being (CR)_0_/29~32, (CR)_23_/25~27, (CR)_88_/41~42, and (CR)_157_/47~50.

### Involvement of reduced ATXN8OS expression with histone modification

**Figure 3:**
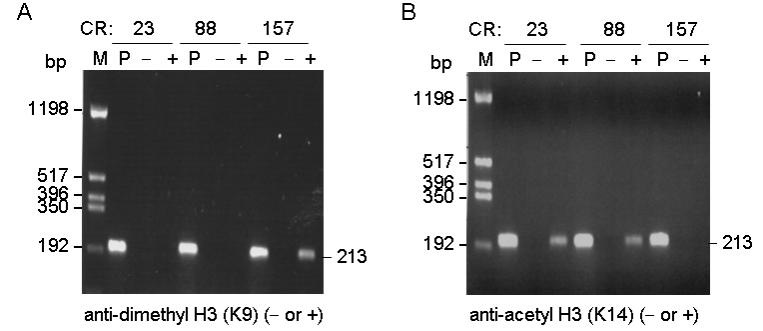
**ATXN8OS CR lines ChIP-PCR assay using anti-dimethyl H3 (K9) and anti-acetyl-histone H3 (K14) antibodies**. ATXN8OS CR cells were sonicated to shear DNA. DNA was then isolated, precipitated with anti-dimethyl H3 (K9) (A) or anti-acetyl-histone H3 (K14) (B) antibody, and used as template for PCR amplification of 213-bp exon B to A-containing fragment. Lanes P represents positive control.

The expression of the ATXN8OS in 0, 23, 88 and 157 CR lines was driven by the same hybrid CMV/TetO_2 _promoter. As CUG triplet repeat expansion in DM1 may alter the adjacent chromatin structure [15], the observed repeat length-dependent repression of ATXN8OS expression may be due to chromatin remodeling. Modifications of the H3 have been shown to induce a change in chromatin activity [23]. To examine the possibility that site-specific H3 modifications might regulate ATXN8OS expression with CUG repeat expansions, ChIP-PCR analysis was performed using primary antibodies anti-dimethyl-H3-K9 (associated with transcriptionally repressed chromatin) and anti-acetyl-H3-K14 (associated with transcriptionally active chromatin). The methylation of H3-K9 in 157 CR cells was evident, but not in 23 or 88 CR cells (Fig. 3A). Also hypoacetylation of H3-K14 was observed in 157 CR cells, but not in 23 or 88 CR cells (Fig. 3B).

### Repeat length-dependent repression of HaloTag gene located next to ATXN8OS cDNA gene

**Figure 4:**
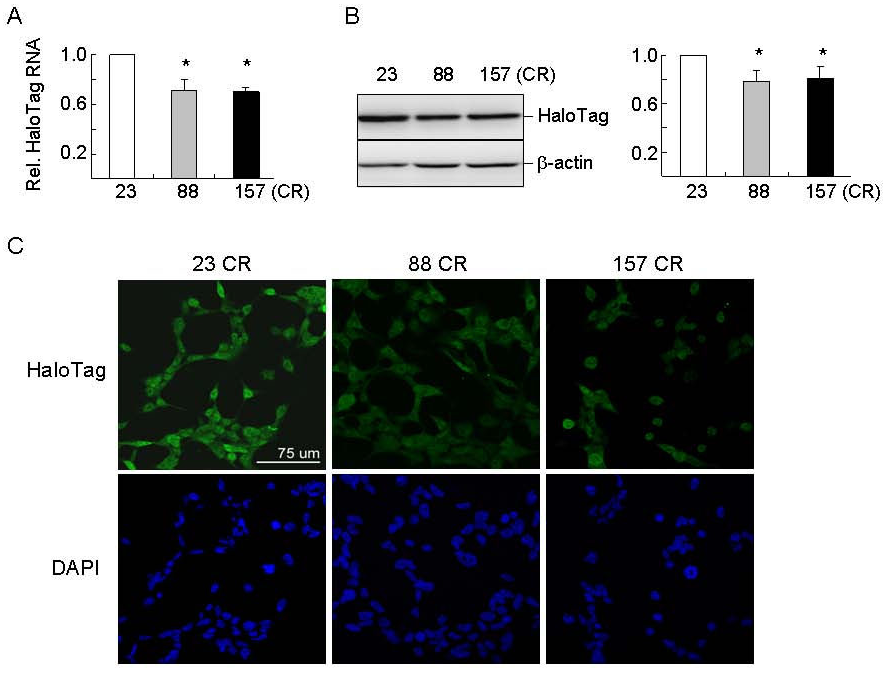
**Expression of HaloTag gene located next to ATXN8OS cDNA gene**. (A) Real-time PCR quantification of HaloTag RNA level relative to endogenous HPRT1 RNA. To normalize, the relative HaloTag RNA in 23 CR cells is set as 1.0. (B) Representative western blot image of CR lines using HaloTag and β-actin antibodies. Levels of HaloTag were normalized with an internal control (β-actin). The relative immunoreactivity of HaloTag is shown in the right panel. For both (A) and (B), data are expressed as the mean ± SEM values from three independent experiments. * indicates *P *< 0.05. (C) Fluorescence microscopy examination after immunostaining using HaloTag antibody (green). Nuclei were counterstained with DAPI (blue).

The cloning vector used to generate ATXN8OS CR lines was modified by placing a HaloTag gene downstream of the ATXN8OS cDNA gene. If chromatin structure was affected in expanded CR lines, reduced expression of HaloTag would be expected. To examine this, HaloTag RNA level relative to endogenous HPRT1 RNA was first quantified by real-time PCR. As shown in Fig. 4A, HaloTag RNA expression in expanded 88 and 157 CR lines was significantly reduced to 70~71% when compared to the normal 23 CR line (*P *< 0.05). To confirm the expression change, proteins were collected and subjected to western blotting with HaloTag and β-actin antibodies. Consistent with the results of real-time PCR quantification, expression levels of HaloTag protein were significantly decreased in ATXN8OS 88 and 157 CR lines as compared to that of 23 CR line (Fig. 4B, 79~81%, *P *< 0.05). Fluorescence microscope examination after immunocytochemical staining using HaloTag antibody also revealed the reduced expression of HaloTag protein (Fig. 4C).

### Increased ATXN8OS transcript stability and ribonuclear foci formation with CUG repeat expansion

**Figure 5:**
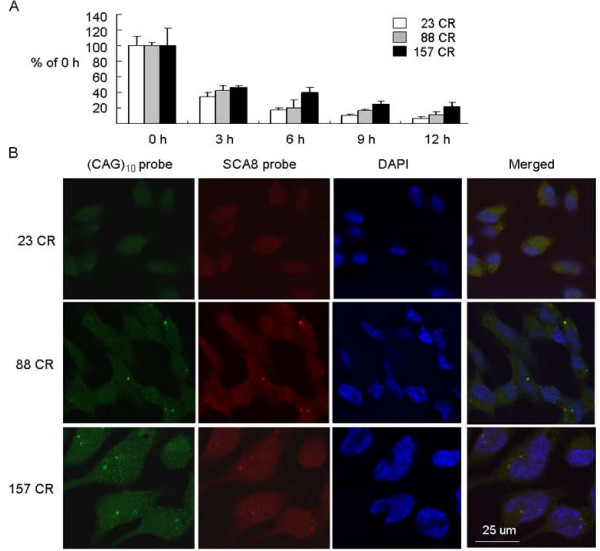
**ATXN8OS transcript stability and ribonuclear foci formation with CUG repeat expansion**. (A) Real-time PCR quantification of ATXN8OS RNA level relative to endogenous HPRT1 RNA following addition of doxycycline for 2 days and actinomycin D treatment for 0, 3, 6, 9 or 12 hours. The relative ATXN8OS RNA in 23, 88 and 157 CR cells at the time of actinomycine D addition (0 h) is set as 100%. (B) Ribonuclear foci formation with ATXN8OS CUG expansion. ATXN8OS 23, 88 and 157 CR cells were grown with doxycycline for 2 days and analyzed by RNA-FISH using a Cy3 labeled (CAG)_10 _(green) or Cy5 labeled ATXN8OS unique sequence (red) oligonucleotide probe. Nuclei were counterstained with DAPI (blue).

To examine the observed repeat length-dependent increase of fold of induction, the effect of repeat length on the stability of ATXN8OS RNA was investigated. The ATXN8OS cells were grown in the presence of doxycycline for 48 h and actinomycin D (1 μg/ml) was added to block transcription of new RNA molecules. The stability of the ATXN8OS RNA was then determined by real-time PCR quantification of the amount of ATXN8OS RNA present in cells harvested at different time points after actinomycin D addition. The amount of ATXN8OS RNA present at the start of the experiment immediately before actinomycin D addition was set to 100%. As shown in Fig. 5A, using HPRT1 mRNA as an internal control, the levels of ATXN8OS mRNA at 12 h after addition of actinomycin D in 23, 88 and 157 CR cells were 7.2%, 12.1% and 22.0%, respectively. Therefore, expanded CR causes stabilization of ATXN8OS mRNA and subsequently reduces RNA decay.

Since the mutant DMPK and JPH3 transcripts accumulated in the nuclei of patient cells and aggregated to form distinct foci [13,19], we investigated whether the expanded ATXN8OS CUG repeats form ribonuclear foci. The ATXN8OS 23, 88 and 157 CR cells were grown in the presence of doxycycline and FISH experiments using a Cy3-labeled (CAG)_10 _oligonucleotide probe was performed two days later. As shown in Fig. 5B, no ribonuclear foci were seen in cells expressing ATXN8OS 23 CR. However, distinct ribonuclear foci, mostly perinuclear, were observed in cells expressing expanded 88 and 157 CR. Similar results were obtained using an oligonucleotide probe specific to ATXN8OS. Since this probe can only bind to the ATXN8OS RNA in single copy, the inability to detect ribonuclear foci with 23 CR is not an artifact of the copy number of the repeats in the 88 or 157 CR cells.

## Discussion

Clinical and genetic studies have shown that SCA8 is a slowly progressive inherited disorder with highly incomplete penetrance, in addition to its rare occurrence of phenotype in individuals carrying a much larger repeat expansion [1,4-7]. Thus the pathogenic mechanisms underlying SCA8 are expected to be complicated. In this study we developed a number of otherwise isogenic human cell lines expressing transcripts with 0~157 CTA/CTG CR. The repeat number in these cell lines was stable and expression of ATXN8OS RNA containing expanded 88 and 157 CR causes an increase in the likelihood of cell death (Fig. 1E). These cells are also sensitive to staurosporine treatment which can induce apoptosis (Fig. 1D). This observation coincides with the reported length-dependent toxicity of untranslated CUG repeats in cell and *Caenorhabditis elegans *experiments [17,19,24]. These cells were used to investigate the possible epigenetic and post-transcriptional controls of the ATXN8OS expression. The implications of the findings in the pathogenesis of SCA8 are discussed as the following.

### Epigenetic changes of ATXN8OS expression

Previously expansions of CTG repeat in myotonic dystrophy and GAA repeat in Friedreich's ataxia conferred variegation of expression on a linked transgene in mice [25]. Silencing was correlated with a decrease in promoter accessibility and was enhanced by the classical position effect variegation modifier heterochromatin protein 1 (HP1), which is able to bind to methylated histone H3-K9 [26]. Elevated levels of histone H3 dimethylated on K9 were also seen in Friedreich's ataxia cells consistent with a repressive chromatin organization [27]. The "Histone Code" hypothesis proposed that amino-terminal extensions of histones are subject to a variety of posttranslational modification [23]. Histone methylation and acetylation are among the best characterized modifications to regulate gene expression through alterations in the chromatin structure. In chromatin domains that are transcriptionally repressed, high levels of histone H3-K9 methylation and H3-K14 hypoacetylation were observed. Therefore, it is possible to predict the transcriptional competence of a particular genomic region by examining the H3 methylation and acetylation patterns. Using ChIP assay, we provided direct evidence of H3-K9 dimethylation and H3-K14 hypoacetylation and repression of ATXN8OS RNA in the 157 CR cells (Fig. 3). As reduced expression of adjacent HaloTag gene was also seen in the 88 CR cells (Fig. 4) independent of H3-K9 dimethylation and H3-K14 hypoacetylation (Fig. 3), DNA methylation or other histone modifications such as arginine methylation and serine/threonine phosphorylation [28] may be responsible for the observed repression of ATXN8OS RNA in the 88 CR cells. Nevertheless the epigenetic change induced by large SCA8 repeat expansions may be one of the genetic factors that suppress the disease symptoms in control individuals carrying large SCA8 repeat expansions. Work is now in progress to examine the roles of HP1 and its associated regulators on the observed repeat-dependent repression of ATXN8OS expression.

### Increased ATXN8OS transcript stability and ribonuclear foci formation

Previously it was reported that expanded CUG repeat transcripts form stable hairpins [29] and muscleblind and MBNL1 increase steady state levels of CUG repeat RNA [30]. The possibility of stable hairpin formation and proteins binding may explain the observation that expanded CR causes stabilization of ATXN8OS mRNA (Fig. 5A), and in turn leads to repeat length-dependent increase in fold of ATXN8OS induction (Fig. 2). In DM1 and DM2, the expanded repeat RNA forms discrete ribonuclear foci to sequester CUG binding proteins and subsequently jeopardize the normal cellular functions of these proteins, which would then lead to abnormal RNA splicing of several genes [31]. Although the induced expression levels of ATXN8OS RNA in these CR cells were low (ranging from 0.659~1.346 compared with endogenous HPRT1), ribonuclear foci were detected in our ATXN8OS 88 or 157 CR cells (Fig. 5B). Most of the RNA foci formed are located near nuclear membrane, which may be compatible with the observation by Koch and colleague that the hairpin structure formed by long CUG repeats (> 44) cannot pass through nucleic pores [32]. The ribonuclear foci observed in the nucleus may also result in transcriptional dysfunction to lead to the disease, as indicated by transcription factors leaching from chromatin by mutant RNA in myotonic dystrophy [33].

## Conclusion

In summary, our data provide evidence of epigenetic and post-transcriptional regulations of the ATXN8OS expression. Although the *in vitro *cell culture study may not truly reflect the pathological events *in vivo*, our study may shed insights into the pathogenesis of this disease.

## Methods

### ATXN8OS cDNA constructs

Human cerebellum polyadenylated RNA (200 ng) (Clontech) was reverse transcribed using the SuperScript™ III reverse transcriptase (Invitrogen). Sense and antisense primers used for amplification of ATXN8OS cDNA were 5'-ATCCTTCACCTGTTGCCT-3' and 5'-GCTTGTGAGGACTGAGAATG-3', respectively. The 1.3-kb full-length, 23 CR [(CTA)_11_(CTG)_12_] containing cDNA (including exons D, C2, C1, B, and A) (Fig. 1A) [34] was cloned into pGEM-T Easy vector (Promega) and sequenced. The cloned ATXN8OS cDNA containing 88 CR was made by replacing a 178 bp *Nla*III-*Afl*II fragment with a 373 bp fragment from the PCR clone of a PD patient [(CTA)_8_CCACTACTGCTACTGCTA(CTG)_74_] [35]. The 88 CR was further expanded to 157 CR [(CTA)_8_CCACTACTGCTACTGCTA(CTG)_67_CTA(CTG)_65_CTA(CTG)_9_] by ligating *Fnu*4HI partially digested fragments. The interruption of the CTG repeat tract by CCA and CTA is similar to that reported seen in SCA8 patients [36]. To construct the clone without combined repeats (0 CR), a *Dra*I site was added to the 5' end of CTA/CTG repeats by site-directed mutagenesis using primer 5'-CCCTGGGTCCTTCATGTTAGAAAACCTGGCTTTAAAA(CTA)_8_C-3' and a 273-bp *Dra*I fragment containing 88 CTA/CTG combined repeats was removed. Then the ATXN8OS cDNA was cloned into the *Not*I site of pcDNA5/FRT/TO vector (Invitrogen) for establishing stably induced ATXN8OS CR cell lines. The pcDNA5/FRT/TO vector used was modified by inserting a 2.3 kb *Bgl*II (blunted)-*Fsp*I fragment containing CMV enhancer/promoter, HaloTag open reading frame and SV40 late poly(A) signal from pHT2 (Promega) at the *Pvu*II site between bovine growth hormone poly(A) signal and Flp recombination target (FRT) site (Fig. 1B).

### Cell culture and ATXN8OS CR cell lines

HEK-293-derived Flp-In™-293 (Invitrogen) cells were cultivated in Dulbecco's modified Eagle's medium containing 10% fetal bovine serum in a 37°C humidified incubator with a 5% CO_2 _atmosphere. The cloned pcDNA5/FRT/TO-ATXN8OS plasmids and vector were used to generate the ATXN8OS CR and vector only cell lines by targeting insertion into Flp-In™-293 cells, according to the supplier's instructions. The repeats in these ATXN8OS cell lines were examined by PCR and sequencing. These cell lines were grown in medium containing 5 μg/ml blasticidin and 100 μg/ml hygromycin. Doxycycline (1 μg/ml) was added to induce ATXN8OS expression. To evaluate the stability of ATXN8OS transcripts, actinomycin D (1 μg/ml) (A1410, Sigma) was added 48 hr after induction for 0, 3, 6, 9 and 12 hr for total RNA preparation.

### Real-time PCR quantification of mRNA

Total RNA was extracted from ATXN8OS CR and vector only cells using the Trizol (Invitrogen). The RNA was DNase (Stratagene) treated, quantified, and reverse-transcribed to cDNA using High Capacity cDNA Reverse Transcription Kit (Applied Biosystems) with random primers. Using ABI PRISM^® ^7000 Sequence Detection System (Applied Biosystems), real-time quantitative PCR was performed on a cDNA amount equivalent to 250 ng total RNA with TaqMan fluorogenic probes Hs01382089-m1 (exon C2 and C1 boundary) for ATXN8OS and 4326321E for HPRT1 (endogenous control) (Applied Biosystems). The amount of HaloTag mRNA was determined by customized Assays-by-Design probe (Forward primer: CCGACGTGGGACGAATGG, Reverse primer: CGGAAGGCCTGGAAGGT, TaqMan^® ^probe: GAATTCGCCCGTGA) (4331348, Applied Biosystems). Fold change was calculated using the formula 2^ΔCt^, ΔC_T _= C_T _(control) - C_T _(target), in which C_T _indicates cycle threshold.

### Cell viability assay

To quantify the cell viability, mitochondrial dehydrogenase activity was evaluated by measuring its cleaving activity of the tetrazolium salt WST-1 (Takara). The cultured cell suspension was seeded into a 96-well plate. After staurosporine (0~50 nM) treatment for one day, PreMix WST-1 was added to cells in a final 1:10 dilution and incubated for 2 hours. The absorbance of the red colored formazan dye cleaved from WST-1 was measured at 450 nm by Microplate Autoreader EL311 (Bio-Tek Instruments Inc.). Reactions were performed in triplicate. Annexin V-positive cells were determined using Annexin V-FITC Apoptosis Detection Kit (Strong Biotech Corp.) according to the supplier's instructions. Each cell line was tested at least 3 times and apoptotic cells quantitatively determined by flow cytometry.

### Chromatin immunoprecipitation (ChIP)-PCR

Cells on five 10 cm dishes were incubated with 1% formaldehyde for 10 min to cross-link histones to DNA. Cells were then washed twice using cold PBS containing protease inhibitors (1 mM phenylmethylsulfonyl fluoride, 1 μg/ml aprotinin and 1 μg/ml pepstain A). The washed cells were scraped and resuspended in lysis buffer (1% SDS, 10 mM EDTA, 50 mM Tris pH 8.1) (Upstate Biotechnology). The resulting lysate was subjected to sonication. The sample was centrifuged to remove cell debris. The volume of the chromatin supernatant was divided into several parts. The first part was used as input (positive) control, and other parts were diluted with ChIP dilution buffer containing protease inhibitors to bring up the volume to 2 ml. The chromatin solution was incubated with 75 μl of a mixture of protein A-agarose/salmon sperm DNA slurry (Upstate Biotechnology) for 30 min at 4°C with agitation to reduce nonspecific background. Following this preclearing procedure, the solution was centrifuged and the supernatant was collected. Five micrograms of anti-dimethyl H3-K9 or anti-acetylated-H3-K14 antibody (Upstate Biotechnology) were added to the chromatin solution and incubated overnight at 4°C with rotation. The resulting immune complexes were collected by addition of 60 μl of protein A-agarose/salmon sperm DNA slurry and incubated at 4°C with rotation for 1 h. The beads were washed several times and the attached immune complexes were eluted with 250 μl elution buffer containing 1% SDS and 0.1 M NaHCO_3_. Cross-links were reversed by the addition of 8 μl 5 M NaCl and incubating the samples at 65°C for 4 hr. The DNA was purified by phenol/chloroform extraction and analyzed by PCR using the primer pair specific to the exons B to A region (5'-CAAACTTCAGAGAGAGAGGG-3' and 5'-CAGAGTTAATCTCTCCGTGG-3', 213 bp fragment) of ATXN8OS cDNA gene (Fig. 1A).

### Western blot analysis

ATXN8OS CR cells were lysed in RIPA buffer (10 mM Tris pH 7.5, 150 mM NaCl, 5 mM EDTA pH 8.0, 0.1% sodium dodecyl sulphate (SDS), 1% deoxycholate, 1% NP-40) containing the protease inhibitor mixture (Sigma). After sonication and sitting on ice for 30 min, the lysates were centrifuged at 13 000 g for 30 min at 4°C. Protein concentrations were determined with the Bio-Rad protein assay kit, using albumin as standards. Laemmli sample buffer was then added to 30 μg of protein and heated in a boiling water bath for 10 min. Equal amounts of protein from each sample were fractionated in a 12% SDS-polyacrylamide gel electrophoresis (PAGE). The fractionated protein samples were transferred onto a nitrocellulose membrane (Schleicher and Schuell), and non-specific binding was blocked in 5% non-fat dry milk for overnight at 4°C. After washing with Tris-buffered saline (TBS), the blots were probed with a 1:1000 dilution of HaloTag antibody (Promega) or a 1:10000 dilution of actin antibody (Chemicon) in TBS/1% bovine serum albumin/0.1% Tween 20 for 1 hr at room temperature with gentle shaking. After extensive washing, the blots were probed with a 1:10000 dilution of goat anti-mouse or goat anti-rabbit IgG conjugated to horseradish peroxidase (Jackson ImmunoResearch). The blots were washed extensively and the protein detected using Immobilon™ western chemiluminescent HRP substrate (Millipore).

### Immunocytochemical staining

ATXN8OS CR cells on coverslips were washed with PBS and fixed in 4% paraformaldehyde in PBS for 10 min, followed by 20 min incubation with 0.1% Triton X-100 in PBS to permeabilize cells, overnight incubation with 0.5% bovine serum albumin in PBS to block non-specific binding. The primary anti-HaloTag antibody, diluted 1:500 in 1% BSA in TBS, was used to stain cells at 4°C overnight. After washing, cells were incubated for 2 hr at room temperature in FITC-conjugated secondary antibody diluted to 1:500 in TBS containing 1% BSA, and washed in PBS. Nuclei were detected using 4'-6-diamidino-2-phenylindole (DAPI). The stained cells were examined after mounted in Vectashield (Vector Laboratories Inc.) using a Leica TCS confocal laser scanning microscope.

### Fluorescent in situ hybridization (FISH)

To examine the ribonuclear foci, cells were grown on coverslips, washed, and fixed for 15 min at room temperature in 4% formaldehyde and 10% acetic acid. After 0.1% Triton X-100 treatment for 10 min, a Cy3-(CAG)_10 _(Operon) or Cy5-CTGCGACTCCGCTGGAAACTCTTCAGCCA (unique toATXN8OS) oligonucleotide probe was added at 37°C for 2 hr for FISH experiments . Nuclei were detected using DAPI (4'-6-diamidino-2-phenylindole). Fluorescent signals are visualized using a Leica TCS confocal laser scanning microscope optimized for simultaneous dual fluorescent imaging.

## Authors' contributions

ICC and HYL carried out the experiment including ATXN8OS CR construct engineering, stable lines establishment and expression studies. GCL carried out the experiment of ChIP-PCR. SHK performed cell viability assay. GJLC supervised the study design. All authors participated in the revising of the manuscript.
